# Effect of Molasses and Dried Orange Pulp as Sheep Dietary Supplementation on Physico-Chemical, Microbiological and Fatty Acid Profile of Comisana Ewe's Milk and Cheese

**DOI:** 10.3389/fnut.2019.00001

**Published:** 2019-02-05

**Authors:** Luigi Liotta, Cinzia L. Randazzo, Nunziatina Russo, Alessandro Zumbo, Ambra Rita Di Rosa, Cinzia Caggia, Vincenzo Chiofalo

**Affiliations:** ^1^Dipartimento di Scienze Veterinarie, Università degli Studi di Messina, Messina, Italy; ^2^Dipartimento di Agricoltura, Alimentazione e Ambiente, Università degli Studi di Catania, Catania, Italy; ^3^Dipartimento di Scienze Chimiche, Biologiche, Farmaceutiche ed Ambientali, Università degli Studi di Messina, Messina, Italy

**Keywords:** citrus by-products, sheep milk yield, milk coagulation properties, sheep cheese, lactobacilli

## Abstract

The use of agro-industrial by-products for ruminant feed represents both an economical and environmental convenient way for reducing waste discharge and waste management costs for food industries. Large amounts of waste from citrus processing industries are available in Sicily, Italy. In the present study, the effect of dried citrus pulp as sheep dietary supplementation was evaluated on physico-chemical, microbiological and fatty acid composition of resulting milk and cheese. Pelleted feed integrated with molasses and blond orange pulp, replacing cane molasses, beet pulp and part of the maize and sunflower in ration, were administrated to ewes as an experimental treatment The experiment involved sixty Comisana breed sheep divided into two groups and two feeding trials (experimental and control). Ewe's milk and cheese samples were collected from January to April and analyzed for physico-chemical, microbiological and fatty acid profile composition. Results suggested that both the experimental milk and cheese were different from the controls. In particular, an increase of experimental milk yield and fat content were registered whilst the cheese samples exhibited a significant decrease of pH values and an increase in fat and protein contents. In addition, an increase of conjugated linoleic acids as well as of the oxidative stability were observed indicating the beneficial effect of dietary supplementation. Furthermore, among the main microbial groups, the experimental and control samples, no differences were detected. However, with the exception of streptococci, which was found higher in experimental cheeses, and staphylococci, which was significantly reduced by experimental feed. Moreover, the application of culture-independent methods highlighted the dominance of *Lactobacillus rhamnosus/casei* group in the experimental cheese, suggesting a driving role of the dietary supplementation in the cheese microbiota composition. The present study demonstrated that the inclusion of citrus by-products in the diet of small dairy ruminants is a promising feeding, which could positively affect milk composition and cheese manufacture.

## Introduction

The use of agro-industrial by-products for ruminant feed is both an economical and environmental way to reduce waste discharge and decrease waste management cost (1). Large amounts of waste from citrus juice extraction are produced in Spain and Sicily, the main citrus fruit producer in the Mediterranean region, accounting for 1.6 million of tons of citrus per year (2). The residues of the juice extraction comprise of peel, pulp, rag and seeds. These components, either individually or in various combinations, are source materials from which by-product feedstuffs can be obtained (3, 4). In Sicily, an average of 34% of citrus fruits are processed into juices leaving about a half of its weight as waste, that presents high energy content, due to high soluble carbohydrate contents, and rapidly degradable Neutral Detergent Fiber (NDF), made up of cellulose and pectin. For its nutrient content can partly replace cereal grains in animal rations, as dehydrated fed, fresh fed or as silage (4) and its use, in formulated feeds, largely depends on its availability and its relative cost-effectiveness, when compared with other alternative raw materials. It has been already demonstrated that these components have fewer negative effects than supplementation with starch or sugar-rich feeds on the rumen ecosystem (5). The high content of pectin involves to a faster rumen fermentation allowing the release of energy for a rapid microbial growth (6) and contributes to create better rumen conditions for fiber fermentation. Its high level of potential degradable dry matter provides a high total digestible nutrient content (7). Moreover, the inclusion of citrus by-products in the diet of small dairy ruminants had some effects on milk yield, composition and properties and on the quality of derived products (8). It has been well established that the addition of orange pulp into the goat's diet affected milk quality and cheese composition resulting in a lower pH and water activity, high fat and NaCl content that contribute to improve the sensory characteristics of derived ripened cheese (1). Although some researches have investigated the effects of different feeding systems on the quality and composition of milk and dairy products, up to now, no information is available on the influence of agro-industrial by-products, such as orange pulp, as sheep dietary supplementation on Sicilian ewe's milk and cheese.

In Sicily, a Southern region of Italy, Comisana sheep milk production is mainly transformed into Protected Denomination of Origin (PDO) Pecorino cheese. Typically, cheese-making is homemade, without addition of any starter cultures and cheese is ripened for at least 6 months before sale. During cheese production and ripening, several microbial and biochemical processes take place that contributing together with milk composition, to the characteristics and the quality of the final product.

The aim of this study was to investigate the effect of replacement of cane molasses and beet pulp, used in conventional fed, with molasses and dried orange pulp on physico-chemical, microbiological and fatty acid composition of Comisana sheep's milk and cheese manufacturing.

## Materials and Methods

### Experimental Design, Animals and Diets

The experimental study was conducted in an organic farm, located in the Enna's province (Sicily, Italy) at an altitude of 600 m above sea level, with about 1,000 sheep of Comisana breed for the production of “Ricotta” and “Pecorino” cheese types. The applied procedures were in compliance with the European guidelines for the care and use of animals in research (Directive 2010/63/EU). The trial ran from 20 days before lambing and was performed on sixty multiparous Comisana ewes at the 5th month of gestation. The animals were randomly divided into two groups of thirty ewes each, homogeneous for age (3–6 years), body weight (49.7 ± 2.7 kg), body condition score (BCS 2.76 ± 0.33) and parity (3 ± 1), named control (C) and experimental (E). During the experimental period a commercial pelleted feed was administered to the control group (C) while a pelleted feed integrated with 4% (DM basis) of molasses and 10% (DM basis) of blond orange pulp were offered daily to ewes of the experimental group (E) in replacing the cane molasses and beet pulp present in the pelleted feed of the control group (Table 1). The mean sugar content of both molasses and orange pulp is reported in Table 2. The pelleted feed of both groups were specifically produced by a feed factory (Di Pasquale Mangimi, Ragusa, Italy) for the trial. The groups were housed in two adjacent free stall pens (60 m^2^/Group) with concrete floor and equipped with feed manger, drinker and covered paddock (100m^2^/Group). The two dietary treatments were isoenergetic and isonitrogenous and were administered for a period of 180 days (from-20 days before at + 160 days post-partum). Their chemical composition, expressed in percentage, is reported in Table 3. Moreover, the sugar composition during the trial, ewes received 500 grams per head per day of pelleted feed and 1.5 kg per head per day of vetch hay divided between morning (05:00 a.m.) and evening (05:00 p.m.) during the milking. In addition, both groups were granted the same natural pasture from 8:00 a.m. (after morning milking) to 2:00 p.m. (before evening milking). The most common botanical families were: Cruciferae (Raphanus spp., Capsella bursapastoris, Sinapis spp., Brassica spp.), Compositae (Carduus spp., Cychorium spp., Calendula aruensis), Graminaceae (Bromus spp.), Leguminosae (Trijiolium spp.), and Malvaceae (Malua neglecta). The above mentioned species were nearly always distributed in very complex mixes.

**Table 1:** Proportion and ingredient of the pelleted complete feed (% of DM).

	**Control**	**Experimental**
Degerminate corn meal	32.50	33.00
Sunflower pellet	29.00	29.10
Beet pulp	10.00	–
Wheat middling	8.80	10.00
Dry orange pulp	–	10.00
Broad bean	7.80	8.70
Lemon molasses	–	4.00
Sugar cane molasses	3.50	–
Corn germ	2.80	2.80
Calcium carbonate	1.90	0.10
Carob pulp	1.10	1.00
Sodium chloride	0.95	0.95
Lysine	0.20	0.20
Magnesium oxide	0.20	0.20
Mineral- vitamin premix	0.15	0.15

**Table 2:** Mean sugar content and composition, expressed as g/100 g, of both orange pulp and molasses.

	**Orange pulp**	**Molasses**
Glucose	2.52	8.80
Fructose	1.97	7.00
Lactose	–	–
Sucrose	5.05	2.35
Maltose	–	–

**Table 3:** Chemical composition of the pelleted complete feed and vetch hay (% as fed).

	**Control**	**Experimental**	**Vetch hay**
Dry matter	89.50	89.30	87.00
Crude protein	18.90	18.93	14.30
Crude fiber	10.82	10.38	25.93
Neutral detergent fiber	28.53	26.83	43.28
Acid detergent fiber	15.99	16.61	30.47
Acid detergent lignin	4.35	4.41	5.68
Starch	30.89	31.45	—
Non-fiber carbohydrate	41.60	43.74	—
Ether extract	2.87	3.02	0.91
Ash	8.06	7.45	8.6
UFL (kg/DM)	0.99	0.99	0.74

*UFL,Milk Forage Unit; energy unit of the INRA system corresponding to 1.70 Mcal of net energy for lactation estimated at maintenance level*.

### Feed, Milk and Cheese Sampling

For each pelleted feed bag, both control and experimental, elementary aliquots were collected, in order to obtain a global sample of 500 g. The same amount of hay was sampled and analyzed following the procedures of the AOAC (9) to determine the concentrations of dry matter (DM, 934.01), ash (942.05), crude protein (CP, 2001.11) and ether extract (EE, 920.39). Concentrations of Neutral Detergent Fiber (NDF) (aNDFom, 2002.04), Acid Detergent Fiber (ADF) (ADFom, 973.18) and Acid Detergent Lignin (ADL) (973.18) were determined according to Van Soest (10), using heat-stable amylase and expressed exclusive of residual ash. Non-fiber carbohydrate (NFC) content was calculated as (100–[CP +EE+ ash+ aNDF]).

The record 0 was taken at 40 ± 5 days lactating (weaning period). After this period individual milk yield of the two daily milking samples (05:00 a.m. and 05:00 p.m.) was recorded and, at the same time, individual milk samples (250 ml) were collected and analyzed to measure fat, protein, casein, lactose, total solids, non-fat solids, urea and titration acidity (Soxhlet-Henkel/ SH), using Fourier Transform InfraRed (Milkoscan FT2, Foss Electric, Sweden), calibrated with appropriate sheep milk standards. Moreover, pH (Orion EA 940), clotting properties, according to the r (clotting time), k_20_ (curd firming time), a_30_ (curd firmness) parameters, using rennet Hansen Standard (200 μl/10 ml of milk) and Formagraph instrument (Foss Electric Hillerod, Denmark) according to A.S.P.A. method (1995) were detected.

Cheese production was performed in a small-scale dairy plant, located in the Enna area of Sicilian region (Italy). According to the protocol of production [(11), p. 295], the raw sheep's milk was processed without adding any starter cultures. The production was carried out in four consecutive manufactures, from January to April 2017, and the ripening was monitored monthly until 60 days. Cheese samples obtained from milk originating from animals fed with control (C) and experimental (E) diets and for each making month, after 60 days of ripening, were collected and subjected to physico-chemical analyses. Overall, eight samples from control (C) and experimental (E) cheeses were obtained. For microbiological analyses, cheese samples were transported to the laboratory of Food Microbiology, at the Department of Agriculture, Food and Environment, University of Catania, in refrigerated conditions and analyzed within 4 h.

### Physico-Chemical Analyses

The pH values of cheese samples were determined by pHmeter (H19017, Microprocessor, Hanna Instruments). Water activity (a_w_), determined by a special apparatus (Aqualab Series 3TE dewpoint electronic water activity meter), with an accuracy of ±0.003, was measured at 21°C on grated cheese (1.5 g), in triplicate. Moreover, cheese samples were analyzed for moisture, fat, protein and salt content, using Near Infrared Spectroscopy in Transmittance (FoodScanTM Dairy Analyser; FOSS, Italy).

### Cholesterol Determination

Cholesterol determination in cheese samples was performed according to the Official Method 994.10-Cholesterol in food- Direct saponification Gas Chromatographic Method (AOAC). In detail, 2 g of test portion were saponified at high temperature with ethanolic KOH solution (40 ml 95% ethanol and 8 ml 50% KOH solution). The unsaponifiable fraction containing cholesterol was extracted with toluene. The cholesterol was derivatizated to trimethylsilyl (TMS) ethers by hexamethyldisilane (HMDS) and trimethylchlorosilane (TMCS). The derivatizeted cholesterol was analyzed by GC-FID (Agilent Technologies 6890 N, Palo Alto, CA, USA) with a split/splitless injector, a flame ionization detector and a fused silica capillary column HP5, 30m × 0.32 mm I.D., 0.25 μm film thickness (Agilent J&W GC columns). The column temperature was programmed at 260°C (60 min). Temperature of the injector and detector was 280°C. Injection volume was 1.0 μl. The carrier gas used was helium (1 ml/min), and the split ratio was 1:50. Cholesterol was identified by comparing the relative retention times with standards from Supelco. Chromatogram peak areas were acquired and calculated by Chemstation software (Agilent). Concentration of cholesterol was calculated by external standard method and expressed as mg/100 g.

### Thiobarbituric Acid-Reactive Substances Determination

The thiobarbituric acid-reactive substances (TBARS) assay was performed as described by Luciano et al. (12). In brief, an aliquot of 2.5 g of cheese was finely minced and homogenized with 12.5 ml of distilled water. Trichloroacetic acid (12.5 ml; 10%, w/v) was added to precipitate proteins. Samples were filtrated and 4 ml of the filtrate were mixed with 1 ml of 0.06 M aqueous thiobarbituric acid. Samples were incubated in a water bath at 80°C for 90 min and the absorbance at 532 nm was measured. The assay was calibrated using standard solutions of 1,1,3,3,-tetra-ethoxypropane in trichloroacetic acid (5% w/v). Results were expressed as mg of malondialdehyde (MDA)/kg of cheese. Three replicates (*n* = 3) were run per sample. TBARS were determined at 0, 10, 20, 30, and 40 days of storage at 4°C.

### Fatty Acids Analysis

Lipids were extracted using a mixture of chloroform/methanol (2:1, v/v), and fatty acids methyl esters of cheese fat were prepared by direct transesterification with sulfuric acid/methanol (1:9, v/v) of a weighed portion (15 mg) of the total lipids and analyzed using the high resolution gas chromatography technique. The fatty acid methyl esters (FAME) were analyzed by GC-FID (Agilent Technologies 6890 N, Palo Alto, CA, USA) with a split/splitless injector, a flame ionization detector and fused silica capillary column Omegawax 250, 30 m × 0.25 mm I.D., 0.25 μm film thickness (Supelco, Bellefonte, PA, USA). The column temperature was programmed as follows: initial isotherm of 160°C (6 min), increment of 3°C/min and a final isotherm of 250°C (30 min). Temperature of the injector and detector was 250°C. Injection volume was 1.0 μl. The used carrier gas was helium (1 ml/min), and the split ratio was 1:50. Fatty acids were identified by comparing the relative retention times of FAME peaks from samples with standards from Supelco. Chromatogram peak areas were acquired and calculated using Chemstation software (Agilent). Concentration of each fatty acid (FA) was expressed as g/100 g, considering 100 g as the summation of the areas of all FAME identified. For each sample, the chromatographic analysis was repeated three times. Regarding the fatty acid profiles of cheese samples, the saturated (SFA), Monousaturated (MUFA), and Polyunsaturated (PUFA) fatty acids were analyzed. In addition, to relate the profile of fatty acids with the risk of cardiovascular disorders, the atherogenicity index (AI) and thrombogenicity (TI) indices were calculated, as proposed by Ulbricht and Southgate (13) through the equation:

(1) AI=[(4×C14:0) + C16:0+C18:0]/(ΣMUFA + ΣPUFA)

(2) $$\begin{array}{l} \text{TI}=(\text{C}14:0+\text{C}16:0+\text{C}18:0)/(0.5\times \text{MUFA}+0.5\times \text{PUFA}\\ \;-\text{n}6+3\times \text{PUFA}-\text{n}3+\text{PUFA}-\text{n}3/\text{PUFA}-\text{n}6) \end{array}$$

AI indicates the relationship between the sum of the main saturated FAs and the main classes of unsaturated FAs (14). TI expresses the tendency to form clots in the blood vessels. It is defined as the relationship between the prothrombogenetic (saturated) and the anti-thrombogenetic fatty acids (MUFAs, PUFAs - n6 and PUFAs - n3) (15).

### Microbiological Analyses

Microbiological analyses of cheese samples at different sampling periods were performed in triplicate. In detail, an aliquot (25 g) of control (C) and experimental (E) cheeses, including the cheese core and the surface, were blended for 3–5 min with sterile saline solution, using a Stomacher Lab Blender 400 (International PBI S.p.A Milan, Italy), and then serially diluted into the same sterile solution. Microbiological counts were performed using the following media and conditions: Plate Count Agar (Sigma, Milan, Italy), incubated at 30°C for 72 h, for mesophilic aerobic bacteria; De Man, Rogosa and Sharp agar (Oxoid, Italy), anaerobically incubated, at 32°C for 48 h for lactobacilli; LM17 agar (Oxoid, Italy), with 0.17 g/l of cycloheximide (Oxoid, Italy), incubated at 32°C and 45°C for lactococci and streptococci, respectively; Violet Red Bile Glucose Agar (Difco, Italy), aerobically incubated at 37°C for 24 h, for *Enterobacteriaceae*; Mannitol Salt Agar, incubated at 37°C for 24–48 h, for staphylococci; Sabouraud Dextrose Agar, incubated at 25°C for 72 h, for yeast and mold. The results were expressed as log_10_ colony forming unit (CFU) per ml or g (log CFU/g-ml), the average of three replicates with standard deviation.

### Total DNA Extraction and PCR-DGGE Analysis

Control and experimental cheese samples, at different sampling times, were collected for direct DNA extraction, as previously reported by Randazzo et al. (16). The concentration and purity of DNA were assessed by measuring optical density using Fluorometer Qubit (Invitrogen, Carlsbad, CA, USA). Extracted DNA was used as template for initial PCR targeting the V2 to V3 region of 16S rDNA, using the universal primers HDA1-GC (CGCCCGGGGCGCGCCCCGGGCGGGGCGGGGGCACGGGGGGACTCCTACGGGAGGCAGCAGT-3′) and HDA2 (5′-GTATTACCGCGGCTGCTGGCAC- 3′). The PCR reaction mixture (25 μl) included PCR master mix 2X (Biotechrabbit), 75 mM Tris-HCl (pH 8.4), 50 mM KCl, 1.5 mM Mg^2+^, 10 mM each of the four deoxynucleoside triphosphates (dNTP), 1.255 U/ml of Taq polymerase, 10 pMol of each primer, and 1 μl of appropriately diluted template DNA. The PCR conditions and DGGE analysis of PCR amplicons were the same as previously described (17).

### Statistical Analysis

Statistical analyses of cheese parameters were performed using the GLM procedure in SAS 9.3 (2011) with “Month of cheese making” and “diet” as fixed factors. When a statistically significant effect (p ≤ 0.05) of the diet was detected, means were compared using *p*-values adjusted, according to the Tukey-Kramer multiple comparisons test. All microbiological statistical analyses were performed using XLSTAT statistical software. The statistical significance among groups at different sampling periods was evaluated by one-way analyses of variance (ANOVA). In addition, the XLSTAT statistical software was used in order to visualize possible correlations between the different microbial groups and physico-chemical parameters of cheese samples.

## Results

### Physico-Chemical Composition of Bulk Milk and Cheese

Physico-chemical composition of bulk milk and experimental and control cheese samples through the lactation period is reported in Table 4. Administration of experimental feed influenced the milk yield that was significantly higher in the E group (*p* ≤ 0.05). The significantly highest fat and protein amount (g/d) (*p* ≤ 0.05) were observed in E group. A significant (*p* ≤ 0.05) increase was observed for clotting time (RCT) in the E group, while no significant differences were observed for curd firming time (k_20_) parameter. Milk urea content was lower for E group, compared to C group (*p* ≤ 0.05), resulting in a better nitrogen utilization in presence of citrus by-products supplementation. The readily fermentable molasses and orange pulp could have helped ewes to use the N diet more efficiently and increased the response. This gives the rumen microbial population a possibility to match the inflow of protein with carbohydrates. Overall, significant differences (*p* ≤ 0.05) between control and experimental samples were detected, indicating that the experimental fed significantly affected all the parameters studied, except a_w_. In detail, the experimental cheese samples presented lower pH values and moisture content during the whole lactation period (Table 4). Significant differences (*p* ≤ 0.05) in salt content were observed, with values ranging from 1.84 g/100g to 2.45 g/100 g. The diet significantly affected cheese fat content, observing a higher content in experimental cheese samples than in control samples. An increase of protein level was also registered in experimental samples, with values ranged from 31.26 g/100 g, in March to 34.52 g/100 g, in February (Table 4).

**Table 4:** Physico-chemical and coagulation parameters of both control and experimental ewe's milk and cheese during at each sampling time.

	**January**		**February**		**March**		**April**		**SEM**	***P*-value**
	**Control**	**Experimental**	**Control**	**Experimental**	**Control**	**Experimental**	**Control**	**Experimental**		
**MILK**										
Yield (g/d)	717.4^a^	1,149^b^	808.8^a^	992.4^b^	573.2^a^	829.2^b^	407.5^a^	523.8^b^	0.65	≤0.01
Fat (%)	6.51^a^	5.58^b^	6.68	6.59	6.01^a^	6,58^b^	6.31^a^	5.95^b^	0.18	≤0.05
Fat (g/d)	46.70^a^	64.11^b^	54.03^a^	65.40^b^	34.45^a^	54.56^b^	25.71^a^	31.17^b^	1.13	≤0.01
Protein (%)	5.20	4.92	5.97	6.09	5.66	5.96	5.73	5.58	0.04	0.44
Protein (g/d)	37.30^a^	56.53^b^	48.28^a^	60.43^b^	32.44^a^	49.42^b^	23.35^a^	29.23^b^	1.15	≤0.01
Casein (%)	5.50	5.18	5.25	5.35	5.81	5.26	4.85	4.86	0.15	0.53
Lactose (%)	4.26	4.37	4.23	4.26	3.93	4.06	3.64	3.94	1.09	0.34
Total solids (%)	18.50^a^	16.50^b^	18.34^a^	17.85^b^	20.07^a^	18.09^b^	17.69	17.19	0.23	≤0.05
Solids non-fat (%)	11.77	11.47	11.45	11.93	11.97	12.31	10.94	10.88	1.10	0.67
Urea (mg/100 ml)	25.55^a^	20.23^b^	21.31^a^	19.73^b^	23.94^a^	19.43^b^	25.79^a^	21.27^b^	0.90	≤0.01
Somatic cell count (log _10_)	4.94	4.97	5.08	5.21	4.60	4.55	4.77	4.84	1.10	0.08
pH	6.72	6.76	6.72	6.71	6.88	6.86	6.78	6.90	0.90	0.10
r (min)	17.30^a^	19.16^b^	16.73^a^	17.89^b^	20.86^a^	21.69^b^	19.80	19.94	0.34	≤0.05
a_30_ (mm)	64.38	64.43	57.96	60.52	55.48^a^	48.66^b^	56.23^a^	48.74^b^	0.42	≤0.05
K_20_ min	1.49	1.51	1.57	1.67	1.57	1.55	1.61	1.64	0.35	0.78
**CHEESE**										
pH	5.71^a^	5.58^b^	5.65^a^	5.56^b^	5.70	5.68	5.76^a^	5.65^b^	0.09	≤0.05
aw	0.90	0.90	0.92	0.91	0.92	0.91	0.91	0.91	0.01	0.15
Moisture (g/100 g)	30.49^a^	28.24^b^	29.96^a^	31.05^b^	33.16^a^	32.53^b^	29.57^a^	28.88^b^	0.02	0.19
Protein (g/100 g)	31.09^a^	31.57^b^	30.10^a^	34.52^b^	29.87^a^	31.26^b^	31.23^a^	33.25^b^	0.06	≤0.05
Fat (g/100 g)	29.54^a^	30.26^b^	29.71^a^	31.24^b^	30.27^a^	31.57^b^	30.28^a^	31.85^b^	0.05	≤0.01
Cholesterol (mg/100 g)	74.10	76.92	93.11^a^	77.67^b^	94.91^a^	74.28^b^	69.06^a^	64.88^b^	0.15	≤0.05
Salt (g/100 g)	2.43	2.45	1.55^a^	1.94^b^	2.11^a^	2.03^b^	1.72^a^	1.84^b^	0.01	≤0.05

a, b *Mean values with different letter in superscript within rows indicates significant differences (p ≤ 0.05) due to fed. SEM, standard error of least square means*.

*r, clotting time, min; a_30_, curd firmness, mm; K_20_, curd firming time, min*.

### Cholesterol and Fatty Acids Content in Cheese Samples

The total cholesterol content, expressed as mg 100/g of fat, was significantly lower in experimental cheese, mostly in February, March and April (−16.58%, −21.74% and −6.05%, respectively, Table 4). The fatty acids (FA) composition and nutritional indices of experimental cheeses are shown in Table 5. In general, fatty acid profiles of cheeses were qualitatively similar, especially regarding to unsaturated fatty acids (MUFA). The saturated fatty acid (SFA) dominated the fatty acid profiles of control samples produced in January and in March, while the replacement of the cane molasses and beet pulp by molasses and blond orange pulp produced a significant decrease of these compounds (*p* ≤ 0.05) in experimental cheese in the same period. The lowest content of SFA was observed in experimental cheese produced in March (58.14%), whereas the highest ratio of these fatty acids was revealed in control samples produced in January (77.04%).

**Table 5:** Cheese fatty acid composition (g/100 g of different group of FA) of control and experimental feed at each sampling time.

	**January**		**February**		**March**		**April**		**SEM**	***P*-value**
	**Control**	**Experimental**	**Control**	**Experimental**	**Control**	**Experimental**	**Control**	**Experimental**		
ΣSFA	77.04^a^	75.18^b^	66.94	66.44	61.13^a^	58.14^b^	64.72	64.45	0.08	0.07
ΣMUFA	18.83^a^	29.30^b^	28.69	28.42	29.95	30.85	27.12	27.37	0.12	0.06
ΣPUFA	3.48	3.53	5.52	5.36	7.23^a^	9.68^b^	6.18	6.14	0.15	0.09
n−3	1.08^a^	0.98^b^	1.67^a^	1.45^b^	2.17	2.18	2.21	2.14	0.01	0.06
n−6	2.40	2.55	3.85^a^	3.92^b^	5.06^a^	5.50^b^	4.04^a^	4.53^b^	0.06	0.08
CLAs	0.65	0.68	0.93^a^	1.14^b^	1.34^a^	1.40^b^	0.98^a^	1.10^b^	0.04	≤0.05
AI	4.49^a^	4.09^b^	2.76^a^	2.86^b^	2.51^a^	2.34^b^	2.89	2.84	0.03	≤0.05
TI	3.52	3.28	2.26^a^	2.44^b^	1.90^a^	1.70^b^	2.18	2.17	0.02	≤0.05

a, b *Mean values with different letter in superscript within rows indicates significant differences (p ≤ 0.05) due to fed. SEM. standard error of least square means*.

*SFA, saturated fatty acid; MUFA, monounsaturated fatty acid; PUFA, polyunsaturated fatty acid; n−3, omega 3 fatty acid series; n−6, omega 6 fatty acid series; CLAs, Conjugated linoleic acids*.

*AI, atherogenic index; TI, thrombogenic index*.

Regarding the MUFAs, nothing different was observed between the two cheese groups, except for control cheese manufactured in January, which showed the lowest registered value. Similar trend of polyunsaturated fatty acid (PUFA) content was observed in the whole experimental period except for experimental sample of March, in which the content was the highest (*p* ≤ 0.05) registered (Table 5). The content of ω-3 fatty acid was significant lower (*p* ≤ 0.05) in experimental cheese produced in January and February, while the content of ω-6 acid in this group was the highest during the whole experimentation period (Table 5). Because of different fatty acid compositions, cheeses were characterized by significant (*p* ≤ 0.05) different health lipid indices, such as index of atherogenicity (AI), index of thrombogenicity (IT) (Table 5). Due to the lowest content of USFAs (22.31%), control cheese produced in January was characterized by the most unfavorable AI and TI values. On the contrary, the health lipid indices reported above were observed in experimental cheese produced in March (AI 2.34; TI 1.70). The conjugated linoleic acids (CLAs) content revealed that the experimental cheeses was high in all months of cheese making and significantly higher than in control cheeses (*p* ≤ 0.05) in February, March and April (Table 5).

### Thiobarbituric Acid-Reactive Substances

The influence of dietary supplementation with citrus by-products on lipid oxidation in cheese is presented in Table 6. The TBARs value of the control samples was higher than that found in experimental samples (Table 6), after 40 days of retention and after the 60 days of ripening (*p* ≤ 0.05).

**Table 6:** Lipid oxidative markers (TBARs) of control and experimental feed at each sampling time (values are expressed in mg MDA/kg) at 60 days of cheese ripening.

**Time**	**January**		**February**		**March**		**April**		**SEM**	***P*-value**
	**Control**	**Experimental**	**Control**	**Experimental**	**Control**	**Experimental**	**Control**	**Experimental**		
0	0.07	0.07	0.05	0.03	0.07	0.06	0.04	0.05	0.03	0.94
10	0.07	0.03	0.05	0.04	0.05	0.05	0.05	0.05	0.01	0.06
20	0.05	0.08	0.06	0.05	0.08	0.07	0.06	0.07	0.03	0.07
30	0.07	0.11	0.08	0.06	0.06	0.06	0.08	0.07	0.07	0.27
40	0.24^a^	0.14^b^	0.12^a^	0.08^b^	0.09^a^	0.05^b^	0.11^a^	0.08^b^	0.06	≤0.05

a, b *Mean values with different letter in superscript within rows indicates significant differences (p ≤ 0.05) due to fed. SEM. standard error of least square means*.

*Time, days of storage at 4°C*.

*Means of six cheeses for each group with five replicates of measurements at the stated time period*.

*TBARs, thiobarbituric acid-reactive substances; MDA, malonaldehyde*.

### Microbiological Data

Results of microbiological analyses carried out on both control and experimental cheese samples at different sampling periods, expressed as an average of three replicates with standard deviation are shown in Table 7. Overall, no difference among the main groups was detected. Lactic acid bacteria (LAB) achieved an average value of about 7.30 log cfu/g in control samples and of 7.40 log cfu/g in experimental cheese, with a slight dominance of thermophilic lactobacilli in both cheese samples. Statistical data revealed that the experimental fed significantly (*p* ≤ 0.05) influenced the level of streptococci, which showed higher values, and the level of staphylococci that was significantly reduced by the addition of citrus by-products (Table 7). Regarding streptococci group, the average value ranged from 6.15 log cfu/g to 7.44 log cfu/g in control cheese. The highest value was revealed in experimental cheese. Staphylococci group suffered a decrease of about 2 log units for coagulase positive staphylococci and of about 1 log unit for coagulase negative in experimental cheese samples, reaching a final value of about 1 log CFU/g. *Enterobacteriaceae*, yeast and mold and total mesophilic bacteria showed similar value in both samples, reaching average values of 4.05, 3.11, and 5.50 log cfu/g, respectively (Table 7).

**Table 7:** Microbial counts expressed as log_10_ CFU/mL or CFU/g and standard deviation (SD) of the main microbial groups detected on sheep's milk and 60 days old cheese samples at different manufactures times.

	**January**		**February**		**March**		**April**		**Mean**	
**Microbial groups**	**Control**	**Experimental**	**Control**	**Experimental**	**Control**	**Experimental**	**Control**	**Experimental**	**Control**	**Experimental**
**MILK**										
Mesophilic lactobacilli	6.15 ± 0.02	6.20 ± 0.03	6.28 ± 0.07	6.20 ± 0.04	6.21 ± 0.05	6.19 ± 0.08	6.14 ± 0.07	6.11 ± 0.06	6.20 ± 0.06	6.18 ± 0.04
Thermophilic lactobacilli	5.54 ± 0.06	5.55 ± 0.06	5.56 ± 0.03	5.51 ± 0.09	5.54 ± 0.10	5.58 ± 0.07	5.55 ± 0.09	5.54 ± 0.04	5.55 ± 0.01	5.55 ± 0.03
Mesophilic lactococci	5.71 ± 0.05	5.68 ± 0.05	5.69 ± 0.04	5.64 ± 0.05	5.60 ± 0.02	5.64 ± 0.08	5.60 ± 0.06	5.65 ± 0.03	5.65 ± 0.06	5.65 ± 0.02
Streptococci	5.19 ± 0.07^a^	5.28 ± 0.03^b^	5.14 ± 0.02^a^	5.33 ± 0.07^b^	5.16 ± 0.07^a^	5.33 ± 0.07^b^	5.18 ± 0.05^a^	5.35 ± 0.02^b^	5.17 ± 0.02^a^	5.32 ± 0.03^b^
*Enterobacteriaceae*	3.16 ± 0.04^b^	3.05 ± 0.05^a^	3.17 ± 0.05^b^	3.00 ± 0.07^a^	3.16 ± 0.07^b^	3.00 ± 0.02^a^	3.14 ± 0.04^b^	3.04 ± 0.06^a^	3.14 ± 0.01^b^	3.02 ± 0.03^a^
Coagulase positive staphylococci	3.10 ± 0.05^b^	2.95 ± 0.05^a^	3.14 ± 0.06^b^	3.00 ± 0.01^a^	3.19 ± 0.01^b^	2.92 ± 0.03^a^	3.19 ± 0.02^b^	2.96 ± 0.03^a^	3.16 ± 0.04^b^	2.96 ± 0.03^a^
Coagulase negative staphylococci	2.01 ± 0.02	1.95 ± 0.01	2.04 ± 0.06	1.93 ± 0.05	2.05 ± 0.03	1.96 ± 0.04	2.08 ± 0.04	1.94 ± 0.09	2.05 ± 0.03	1.95 ± 0.01
Yeast and molds	3.14 ± 0.02^c^	2.91 ± 0.04^a^	3.05 ± 0.07^b^	2.90 ± 0.02^a^	3.14 ± 0.03^c^	2.80 ± 0.08^a^	3.09 ± 0.07^b^	2.94 ± 0.02^a^	3.11 ± 0.04^b^	2.89 ± 0.06^a^
Total mesophilic bacteria	5.06 ± 0.06	5.00 ± 0.08	5.05 ± 0.05	5.08 ± 0.06	5.06 ± 0.04	5.02 ± 0.04	5.04 ± 0.06	5.05 ± 0.05	5.05 ± 0.01	5.04 ± 0.04
**CHEESE**										
Mesophilic lactobacilli	7.29 ± 0.03	7.35 ± 0.01	7.25 ± 0.02	7.18 ± 0.03	7.26 ± 0.04	7.30 ± 0.02	7.39 ± 0.06	7.52 ± 0.09	7.30 ± 0.06	7.34 ± 0.14
Thermophilic lactobacilli	7.39 ± 0.04	7.50 ± 0.08	7.47 ± 0.03	7.72 ± 0.07	7.44 ± 0.06	7.46 ± 0.04	7.28 ± 0.04	7.38 ± 0.07	7.40 ± 0.08	7.52 ± 0.15
Mesophilic lactococci	7.40 ± 0.07	7.45 ± 0.12	7.25 ± 0.04	7.21 ± 0.06	7.26 ± 0.03	7.28 ± 0.07	7.38 ± 0.05	7.40 ± 0.04	7.32 ± 0.08	7.34 ± 0.11
Streptococci	6.17 ± 0.04^a^	7.34 ± 0.05^b^	6.07 ± 0.06^a^	7.38 ± 0.04^b^	6.13 ± 0.04^a^	7.51 ± 0.05^c^	6.24 ± 0.02^a^	7.53 ± 0.08^c^	6.15 ± 0.08^a^	7.44 ± 0.09^b^
*Enterobacteriaceae*	4.02 ± 0.05	4.00 ± 0.05	4.00 ± 0.12	4.17 ± 0.05	4.04 ± 0.04	4.00 ± 0.05	4.06 ± 0.04	4.11 ± 0.04	4.03 ± 0.03	4.07 ± 0.08
Coagulase positive staphylococci	3.40 ± 0.05^d^	1.25 ± 0.03^b^	3.32 ± 0.03^c^	1.03 ± 0.06^a^	3.43 ± 0.02^d^	1.03 ± 0.02^a^	3.36 ± 0.03^c^	1.04 ± 0.01^a^	3.38 ± 0.05^c^	1.09 ± 0.11^a^
Coagulase negative staphylococci	2.21 ± 0.06^b^	1.00 ± 0.01^a^	2.20 ± 0.03^b^	1.01 ± 0.07^a^	2.26 ± 0.03^b^	1.25 ± 0.04^a^	2.15 ± 0.03^b^	1.06 ± 0.02^a^	2.21 ± 0.05^b^	1.08 ± 0.12^a^
Yeast and molds	3.05 ± 0.07	3.00 ± 0.02	3.10 ± 0.04	3.11 ± 0.08	3.24 ± 0.06	3.13 ± 0.05	3.09 ± 0.03	3.14 ± 0.06	3.12 ± 0.08	3.10 ± 0.06
Total mesophilic bacteria	5.57 ± 0.01	5.40 ± 0.05	5.60 ± 0.03	5.42 ± 0.09	5.47 ± 0.02	5.39 ± 0.04	5.52 ± 0.03	5.64 ± 0.06	5.54 ± 0.08	5.46 ± 0.12

a, b, c, d *Mean values with different letter in superscript within rows indicates significant differences (p ≤ 0.05)*.

### PCR-DGGE

**Figure 1:**
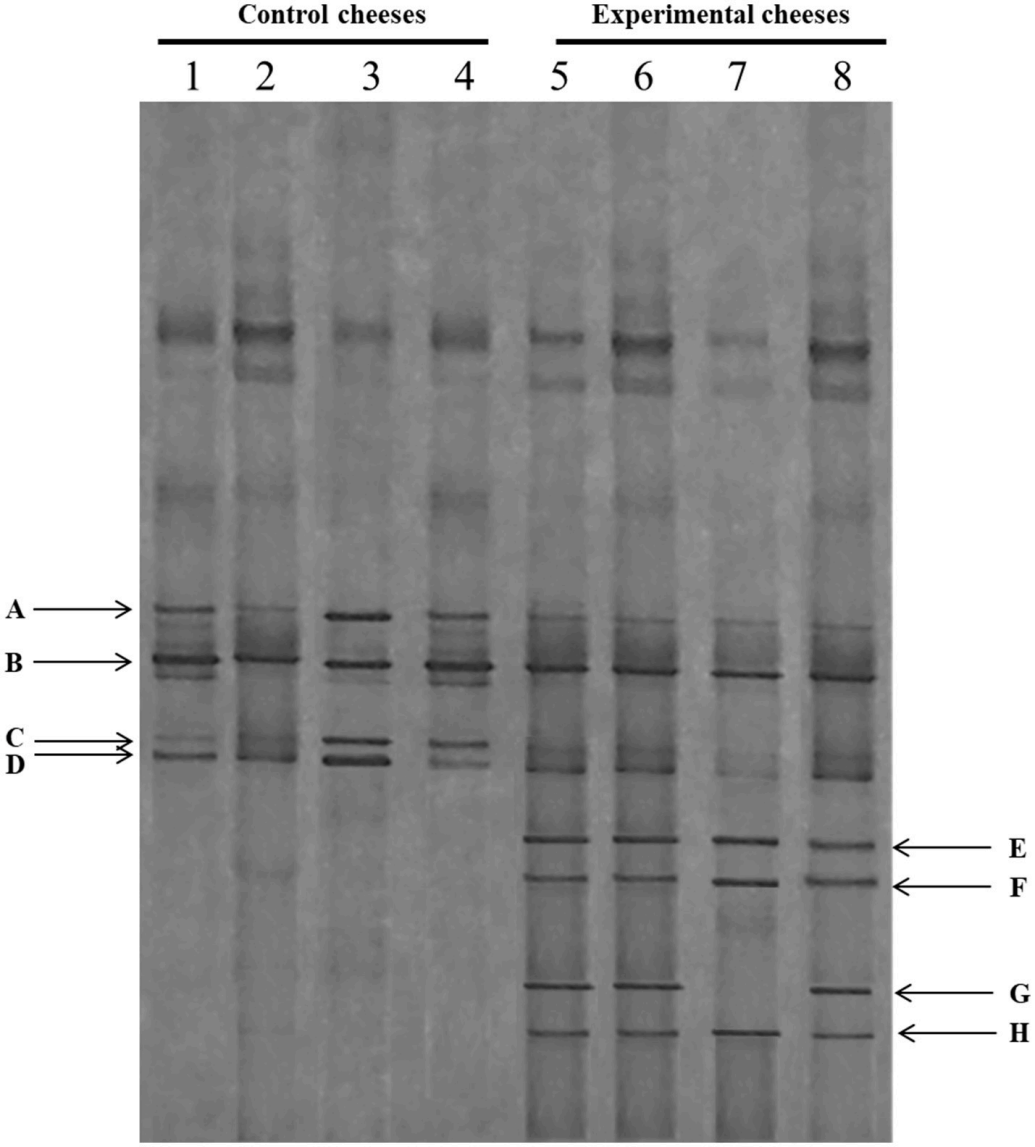
DGGE profiles of the V2-V3 region of the 16S rRNA gene of control (C) and experimental cheese (E) samples. Lanes 1–4: C samples at different manufactures time; lanes 4–8: E samples at different manufactures time. A: *Enterococcus faecalis;* B: *Streptococcus thermophilus;* C: *Enterococcus durans*; D: *Enterococcus faecium*; E, F, H: *Lactobacillus rhamnosus/casei* group; G: *Lactobacillus fermentum*.

PCR-DGGE results of the 16S rDNA of bacterial population of control and experimental cheeses at different sampling periods are reported in Figure 1. In order to identify the dominant species, the DGGE profiles generated from control and experimental cheeses were compared to those of strains, previously isolated from Pecorino cheese and identified (data not shown). Overall, the microbial community of both cheese samples remained quite stable with several bands in common. In detail, the bands A and B, dominated all cheese samples and showed an identical profile of *Enterococcus faecalis* and *Streptococcus thermophilus*, respectively (Figure 1). In addition, the weak bands C and D were detected in all cheese samples analyzed, and were even more pronounced in the control cheese. They showed identical profile to *Enterococcus durans* and *Enterococcus faecium*, respectively. It is interesting to note the appearance of several bands, E, F, and H only in the experimental cheeses, which were identified as *Lactobacillus rhamnosus/casei* group, with the exception of the band G, correspondent to *Lactobacillus fermentum* species, which was not detected in the samples produced in March (line 7).

## Discussion

The present study investigated the effect of citrus by products as sheep dietary supplementation on physico-chemical, microbiological and fatty acid composition of Comisana sheep's milk and cheese. Despite several studies have investigated the effect of including in various forms olive by-products (18–20) or tomato wastes (21–23) in the diet of ruminants, to our knowledge there is no information available on feeding dairy Comisana sheep with fodder made from a mixture of pelleted feed integrated with molasses and blond orange pulp. Overall, results showed that experimental fed significantly affected the physico-chemical and microbiological parameters, in contrast with findings reported by Fegeros et al. (24) who, evaluating the use of dry citrus pulp (11%) in the ration of dairy ewes, did not observe any significant effect on the composition of milk. Results of the present study firstly demonstrated that the experimental feeding system significantly decreased pH and moisture of the experimental cheeses. The low pH value could be related to the high occurrence of LAB at 60 days of ripening. It is noteworthy that LAB produce large amount of lactic acid, as a consequence of their metabolism (25), which could explain the pH lowering in the experimental cheese. In addition, the pH decrease could be related to the loss of colloidal calcium phosphate from casein submicelles with a progressive dissociation of submicelles into smaller casein aggregates, leading to a greater proteolytic effect (26). In cheese, proteolysis can have a significant effect on several attributes, including development of texture and flavor (27, 28), which highly affect the overall acceptability of the final products. In the present study, it clearly showed that the experimental feed improved the microbial diversity in derived cheese samples, particularly in terms of high occurrence of lactobacilli. In fact, the application of culture-independent approach, such as DGGE, lead to the detection of *L. rhamnosus/casei* group and *L. fermentum* species in the experimental 60 days ripened cheese, confirming their importance in the cheese proteolysis and lipolysis. It is already well established that the peptidase activity of lactobacilli could increase the levels of free amino acids (FFA), affecting the development of flavor properties of cheese (28).

One of the most important results of the present study concerns the enhancement of the daily milk production and the fat content in experimental samples, in accordance with a recent study (29). It is well known that milk fat quality is affected by the composition of feed diets (30), and that feed ingredients influence the composition of milk fatty acids (FA) (31). In this context, nutritional quality is becoming a major issue in food choices because of rising consumer awareness of the link between diet and health, increasing market demand for functional foods. An overall improvement of the nutritional composition of experimental cheese, compared to controls was achieved. Our data showed a significant increase of CLAs, which are considered bioactive compounds, with beneficial effect on human health, as demonstrated in several studies (32, 33). Yang et al. (34) reviewed the array of benefits associated with CLA, including positive effects on immune function and protective effects against cancer, obesity, diabetes, and atherosclerosis in animal and human cell line studies. However, no significant difference was revealed for MUFA, PUFA, n-3 and n-6 for the whole trial period, even if an increasing trend was observed in several sampling time. The nutritional composition of the experimental cheese was also improved by the low thrombogenicity anti-atherogenic index scores, which enhanced the health benefit of the product. In addition, our data clearly demonstrated a positive effect of the experimental feed on cholesterol content, which could be related to the antioxidant properties of citrus by-product (35). The reduction of cholesterol in foods is of nutritional interest because its high level in human plasma is associated with an increasing risk of cardiovascular disease (36, 37). The lipid oxidation in cheese, expressed by the TBARs value, was higher in control cheese, and this could lead to the formation of flavor defects and nutritional quality losses (38, 39).

Citrus fruit waste is rich in biologically active compounds, including natural antioxidants, such as phenolic acids and flavonoids (40), that show several therapeutic properties and act as antioxidant, anticancer, antitumor, and anti-inflammatory agents. Hence, the inclusion of citrus by-products in the diets for dairy ewes could increase the intake of total polyphenols, which modifying rumen fermentation (30, 41). In the present study, it has been found that LAB population clearly dominated, while staphylococci were detected at lower level in the experimental cheese. This is probably due both to the high acidification rate of LAB population and to the antimicrobial activity of polyphenols.

## Conclusion

Citrus by-products are cheap raw material, widely available in Mediterranean countries and their potential as a feed ingredient is promising especially for sheep and cattle which are the dominant farm animal species in the area. In the present study the dietary mixture of pelleted feed integrated with molasses and blond orange pulp is a promising strategy in the diet of lactating sheep, which could improve milk and cheese nutritional quality and, therefore, enhancing the health properties of final products.

## Author Contributions

LL, NR, AZ, and ARDR performed the experiments. LL and CR analyzed data. LL, CR and CC wrote the manuscript. LL, CR, CC and VC designed the study and contributed to data interpretation. All authors revised the manuscript.

### Conflict of Interest Statement

The authors declare that the research was conducted in the absence of any commercial or financial relationships that could be construed as a potential conflict of interest.

## References

[1] Salvador A Igual M Contreras C Martines-Navarrete N del Mar Camacho M Effect of the inclusion of citrus pulp in the diet of goats on cheeses characteristics. Small Rumin Res. (2014) 121:361–7. 10.1016/j.smallrumres.2014.06.012

[2] ISTAT Istituto Nazionale di Statistica. Fonte ISTAT (2017). Available online at: http://dati.istat.it/.

[3] Lashkary S Taghizadeh A Seifdavati J Salem AZM Qualitative characteristics, microbial populations and nutritive values of orange pulp ensiled with nitrogen supplementation. Slovak J Anim Sci. (2014) 47:90–9.

[4] Todaro M Alabiso M Scatassa ML Di Grigoli A Mazza F Maniaci G Effect of the inclusion of fresh lemon pulp in the diet of lactating ewes on the properties of milk and cheese. Anim Feed Sci Technol. (2017) 225:213–23. 10.1016/j.anifeedsci.2017.02.003

[5] Bampidis VA Robinson PH Citrus by-products as ruminant feeds. A review. Anim Feed Sci Technol. (2006) 128:175–217. 10.1016/j.anifeedsci.2005.12.002

[6] Hall MB Pell AN Chase LE Characteristics of neutral detergent-soluble fiber fermentation by mixed ruminal microbes. Anim Feed Sci Technol. (1988) 70:23–39. 10.1016/S0377-8401(97)00068-0

[7] De Peters EJ Fadel JG Arosena A Digestion kinetics of neutral detergent fiber and chemical composition within some selected by-product feedstuffs. Anim Feed Sci Technol. (1997) 67:127–40. 10.1016/0377-8401(96)01145-5

[8] Jaramillo DP Garcia T Buffa M Rodriguez M Guamis B Trujillo A. Effect of the inclusion of whole citrus in the ration of lactating ewes on the properties of milk and cheese characteristics during ripening. J Dairy Sci. (2009) 92:469–76. 10.3168/jds.2008-1293 19164656

[9] AOAC Official Methods of Analysis of Association of Official Analytical Chemists. 18th Ed Washington, DC: AOAC (2005).

[10] Van Soest PJ Nutritional Ecology of the Ruminant. 2nd Ed Ithaca, NY: Cornell University Press (1994).

[11] GURI Riconoscimento delle denominazioni circa i metodi di lavorazione, caratteristiche merceologiche e zone di produzione dei formaggi. Official Gazette Italian Repub. 295. (1955). Available online at: http://www.normattiva.it/uri-res/N2Ls?urn:nir:presidente.repubblica:decreto:1955;1269

[12] Luciano G Vasta V Monahan FJ López-Andrés P Biondi L Lanza M Antioxidant status, colour stability and myoglobin resistance to oxidation of longissimus dorsi muscle from lambs fed a tannin-containing diet. Food Chem. (2011) 124:1036–42. 10.1016/j.foodchem.2010.07.070

[13] Ulbricht TLV Southgate DAT. Coronary heart disease: seven dietary factors. Lancet (1991) 338:985–92. 10.1016/0140-6736(91)91846-M 1681350

[14] Prandini A Sigolo S Piva G A comparative study of fatty acid composition and CLA concentration in commercial cheeses. J Food Compost Anal. (2011) 24:55–61. 10.1016/j.jfca.2010.04.004

[15] Garaffo MA Vassallo-Agius R Nengas Y Lembo E Rando R Fatty acids profile, atherogenic (IA) and thrombogenic (IT) health lipid indices, of raw roe of Blue Fin Tuna (*Thunnus* thynnus L.) and their salted product “bottarga”. Food Nutr Sci. (2011) 2:736–43. 10.4236/fns.2011.27101

[16] Randazzo CL Torriani S Akkermans ADL de Vos WM Vaughan EE. Diversity, dynamics, and activity of bacterial communities during production of an artisanal Sicilian cheese as evaluated by 16S rRNA analysis. Appl Environ Microbiol. (2002) 68:1882–92. 10.1128/AEM.68.4.1882-1892.2002 11916708 PMC123848

[17] Pino A Van Hoorde K Pitino I Russo N Carpino S Caggia C. Survival of potential probiotic lactobacilli used as adjunct cultures on Pecorino Siciliano cheese ripening and passage through the gastrointestinal tract of healthy volunteers. Int J Food Microbiol. (2017) 252:42–52. 10.1016/j.ijfoodmicro.2017.04.012 28458191

[18] Abbeddou S Rischkowsky B Hilali MED Haylani M Hess HD Kreuzer M. Supplementing diets of Awassi ewes with olive cake and tomato pomace: on-farm recovery of effects on yield, composition and fatty acid profile of the milk. Trop Anim Health Prod. (2014) 47:145–52. 10.1007/s11250-014-0699-x 25326442

[19] Chiofalo B Liotta L Zumbo A Chiofalo V Administration of olive cake for ewe feeding: effect on milk yield and composition. Small Rum Res. (2004) 55:169–76. 10.1016/j.smallrumres.2003.12.011

[20] Molina-Alcaide E Morales-García EY Martín-García AI Ben Salem H Nefzaoui A Sanz-Sampelayo MR. Effects of partial replacement of concentrate with feed blocks on nutrient utilization, microbial N flow, and milk yield and composition in goats. J Dairy Sci. (2010) 93:2076–87. 10.3168/jds.2009-2628 20412923

[21] Ben Salem H Znaidi IEA Partial replacement of concentrate with tomato pulp and olive cake-based feed blocks as supplements for lambs fed wheat straw. Anim Feed Sci Technol. (2008) 147:206–22. 10.1016/j.anifeedsci.2007.09.019

[22] Romero-Huelva M Molina-Alcaide E Nutrient utilization, ruminal fermentation, microbial N flow, microbial abundances and methane emissions in goats fed diets including tomato and cucumber waste fruits. J Anim Sci. (2012) 91:914–23. 10.2527/jas.2012-5212 23243169

[23] Romero-Huelva M Ramirez-Fenosa MA Planelles-Gonzales R Garcia-Casado P Molina-Alcaide E. Can by-products replace conventional ingredients in concentrate of dairy goat diet? J Dairy Sci. (2017) 100:4500–12. 10.3168/jds.2016-11766 28342612

[24] Fegeros K Zervas G Stamouli S Apostolaki E. Nutritive value of dried citrus pulp and its effect on milk yield and milk composition of lactating ewes. J Dairy Sci. (1995) 78:1116–21. 10.3168/jds.S0022-0302(95)76728-5 7622722

[25] Caridi A Micari P Caparra P Cufari A Sarullo V Ripening and seasonal changes in microbial groups and in physico-chemical properties of the ewes' cheese Pecorino del Poro. Int Dairy J. (2003) 13:191–200. 10.1016/S0958-6946(02)00157-7

[26] Upreti P McKay LL Metzger LE. Influence of calcium and phosphorus, lactose, and salt-to-moisture ratio on cheddar cheese quality: changes in residual sugars and water-soluble organic acids during ripening. J Dairy Sci. (2006) 89:429–43. 10.3168/jds.S0022-0302(06)72107-5 16428613

[27] Fenelon MA Guinee TP Primary proteolysis and textural changes during ripening in Cheddar cheeses manufactured to different fat contents. Int Dairy J. (2000) 10:151–8. 10.1016/S0958-6946(00)00040-6

[28] McSweeney PHL Sousa MJ Biochemical pathways for the production of flavour compounds in cheese during ripening: a review. Le Lait (2000) 80:293–324. 10.1051/lait:2000127

[29] Hilali MHD Rischkowsky BA IIniguez L Mayer H Schreiner M Changes in the milk fatty acid pro le of Awassi sheep in response to supplementation with agro-industrial by-products. Small Rum Res. (2018) 166:93–100. 10.1016/j.smallrumres.2018.06.001

[30] Vasta V Nudda A Cannas A Lanza M Priolo A Alternative feed resources and their effects on the quality of meat and milk from small ruminants. Anim Feed Sci Technol. (2008) 147:223–46. 10.1016/j.anifeedsci.2007.09.020

[31] Buccioni A Minieri S Conte G Benvenuti D Pezzati A Antongiovanni M Changes in conjugated linoleic acid and C18:1 isomers profile during ripening of Pecorino Toscano cheese produced with raw milk. Ital J Anim Sci. (2012) 11:426–30. 10.4081/ijas.2012.e75

[32] Recio I de la Fuente MA Juárez M Ramos M Bioactive components in sheep milk. In: Park YW, editor. Bioactive Components in Milk and Dairy Products. New Jersey, NJ: Wiley-Blackwell (2009). p. 83–104.

[33] Wahle KWJ Heys SD Rotondo D Conjugated linoleic acids: are they beneficial or detrimental to health? Prog Lipid Res. (2004) 43:553–87. 10.1016/j.plipres.2004.08.002 15522764

[34] Yang B Chen H Stanton C Ross RP Zhang H Chen YQ Review of the roles of conjugated linoleic acid in health and disease. J Func Food (2015) 15:314–25. 10.1016/j.jff.2015.03.050

[35] Oluremi OIA Ngi J Andrew IA Phytonutrients in citrus fruit peel meal and nutritional implication for livestock production. Liv Res Rur Dev. (2007) 19:1–8.

[36] Gómez-Cortés P Viturro E Juárez M de la Fuente MA Alternative to decrease cholesterol in sheep milk cheeses. Food Chem. (2015) 88:325–7. 10.1016/j.foodchem.2015.05.012 26041199

[37] Karimi R Mortazavian AM Karami M. Incorporation of *Lactobacillus casei* in Iranian ultrafiltered Feta cheese made by partial replacement of NaCl with KCl. J Dairy Sci. (2012) 95:4209–22. 10.3168/jds.2011-4872 22818434

[38] Botsoglou NA Fletouris DJ Papageorgiou GE Vassilopoulos VN Mantis AJ Trakatellis AG Rapid, sensitive, and specific thiobarbituric acid method for measuring lipid peroxidation in animal tissue, food, and feedstuff samples. J Agric Food Chem. (1994) 42:1931–7. 10.1021/jf00045a019

[39] Fox PF Guinee TP Cogan TM McSweeney PLH Cheese rheology and texture. In: Fox PF Guinee TP Cogan TM McSwee- ney PLH, editors. Fundamentals of Cheese Science. Gaithersburg, MD: Aspen Publishers Inc (2000). p. 305–40.

[40] Li BB Smith B Hossain MM Extraction of phenolics from citrus peels: II. Enzyme-assisted extraction method. Separ Purif Technol. (2006) 48:189–96. 10.1016/j.seppur.2005.07.019

[41] Newbold CJ McIntosh FM Williams P Losa R Wallace RJ Effects of a specific blend of essential oil compounds on rumen fermentation. Anim Feed Sci Technol. (2004) 114:105–12. 10.1016/j.anifeedsci.2003.12.006

